# Supplementary material for: Characterization of the dynamics and variability of neuronal subtype responses during growth, degrowth, and regeneration of Nematostella vectensis
Source: BMC Biol. 2021 May 18;19:104. doi: 10.1186/s12915-021-01038-9 (PMC8128482; doi:10.1186/s12915-021-01038-9)
Supplement: Supplementary file 1 — Additional file 1: Table S1. Average number of tentacular neurons per tentacle in different sized animals. Table S2. Number of tentacular neurons in each tentacle of animals of different sizes. Table S3. Number of longitudinal neurons counted in each longitudinal track. Table S4. Number of tripolar neurons counted in each radial segment. [file 12915_2021_1038_MOESM1_ESM.pdf]

Table S1: Average number of tentacular neurons per tentacle in different sized animals.

Size category	N (12 tentacles/animal)	Mean number of neurons/tentacle*	STDEV	SEM
Small	3 animals	45.22	25.17	4.20
Medium	3 animals	71.94	30.57	5.10
Medium-large	3 animals	84.92	57.13	9.52
Large	3 animals	88.88	44.92	7.49

*Averages derived from data in supplementary table 2.

Table S2: Number of tentacular neurons in each tentacle of animals of different sizes.

	Size category	T1	T2	T3	T4	T5	T6	T7	T8	T9	T10	T11	T12	Min	Max
1	Small	59	40	20	39	12	84	72	96	31	63	40	101	20	101
2	Small	33	47	46	19	12	53	57	39	37	82	41	9	9	82
3	Small	13	9	44	83	21	75	60	55	19	38	54	25	9	83
4	Medium	21	66	47	132	43	81	112	90	25	78	93	34	21	132
5	Medium	92	87	64	156	65	62	52	71	88	48	70	81	48	156
6	Medium	86	35	91	107	72	39	56	123	81	64	28	50	28	123
7	Medium-large	34	20	19	49	10	58	40	5	53	42	25	89	5	89
8	Medium-large	131	111	71	51	189	126	99	201	211	142	168	101	51	211
9	Medium-large	32	87	64	107	28	149	186	104	60	48	77	70	32	186
10	Large	167	182	84	53	99	182	121	101	94	145	171	158	53	182
11	Large	66	42	31	17	70	59	18	89	60	67	71	57	17	89
12	Large	129	51	102	104	50	89	99	51	34	102	96	89	34	129

T= tentacle. All animals that were counted had 12 total tentacles.

Table S3: Number of longitudinal neurons counted in each longitudinal track.

	Neurons counted in each of eight longitudinal tracks								Mean	± SE
Individual 1	12	10	10	12	10	9	14	14	11.38	0.68
Individual 2	18	18	24	18	20	23	16	17	19.25	1.01
Individual 3	41	39	40	35	38	38			38.5	0.85
Individual 4	39	41	43	43	40	43	44	44	42.13	0.67
Individual 5	47	46	49	45	47	46	44	42	45.75	0.71
Individual 6	50	57	51	47	51	48	43	47	49.25	1.45
Individual 7	55	54	51	54	53				53.4	0.68
Individual 8	70	74	74	61	69	69	64	59	67.5	1.99
Individual 9	63	64	63	69	61	58	69	54	62.63	1.80
Individual 10	69	67	75	74	67	65	64	67	68.5	1.41

Blank cells show instances where longitudinal neuron counts were not done.

Table S4: Number of tripolar neurons counted in each radial segment.

	Neurons counted in each of eight radial segments								Mean	± SE
Individual 1	9	10	8	10	13	5	12	14	10.13	1.03
Individual 2	16	14	22	16	21	18	17	15	17.38	1
Individual 3	43	40	47	40					42.5	1.66
Individual 4	44	48	40	44	47	47			45	1.21
Individual 5	65	50	70	57	45				57.4	4.61
Individual 6	52	55	51	57	51	48	43	47	50.5	1.58
Individual 7	54	55	60	55	51				55	1.45
Individual 8	95	75	95	96	85	96	99	100	92.63	2.98
Individual 9	87	86	85	75	76	75			80.67	2.4
Individual 10	83	92	70	78	77	79	75		79.14	2.61

Blank cells show instances where tripolar neuron counts were not done.
