# Supplementary figures and images for: Characterization of the dynamics and variability of neuronal subtype responses during growth, degrowth, and regeneration of Nematostella vectensis
Source: BMC Biol. 2021 May 18;19:104. doi: 10.1186/s12915-021-01038-9 (PMC8128482; doi:10.1186/s12915-021-01038-9)

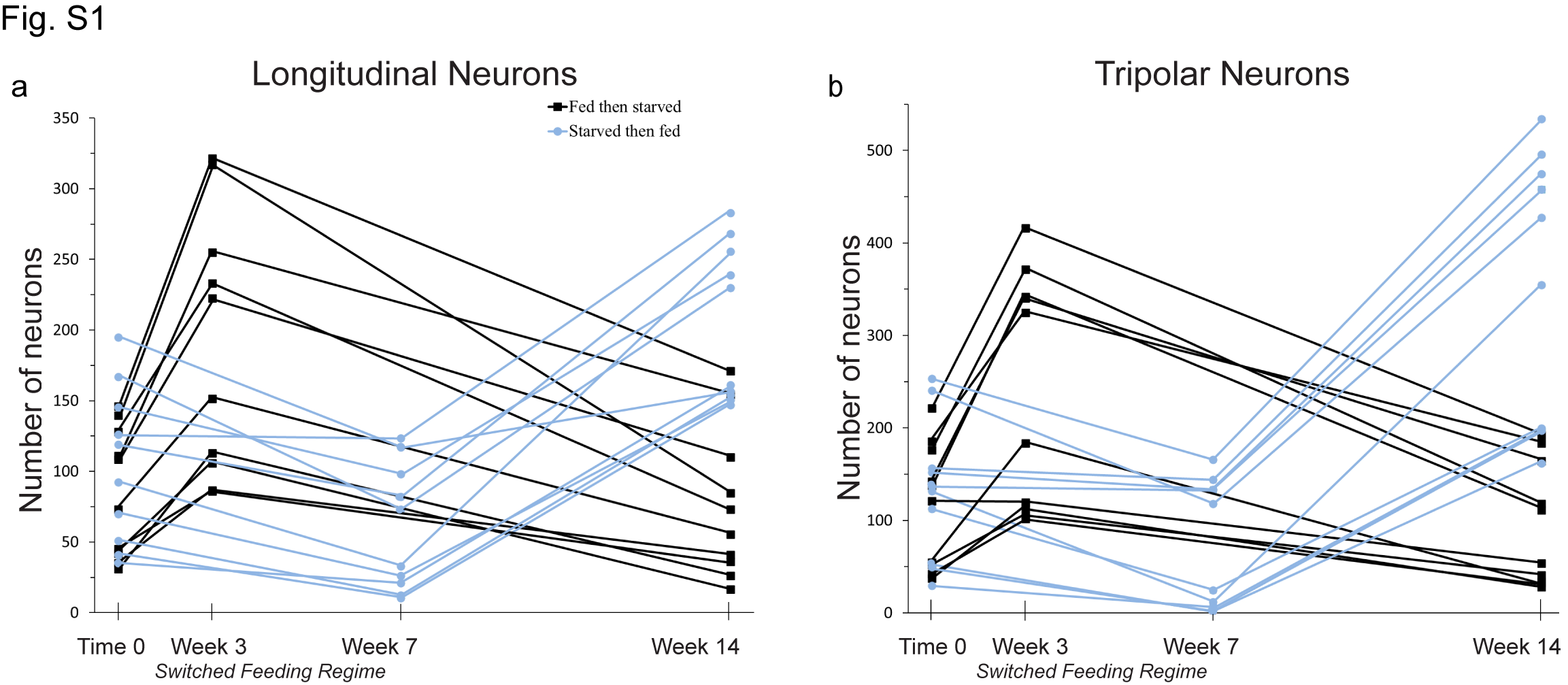

Supplement: Supplementary file 2 — Additional file 2: Fig. S1. Individual longitudinal and tripolar neuron data from the feed/starve experiment. a, b Neurons were quantified in twenty individuals (10/treatment) at the start, time of feeding regime switch, and end of the feed/starve experiment. These data were used to determine the average responses of fed-then-starved and starved-then-fed animals, which are shown in Fig. 2. See Additional file 15 for statistical analyses. [file 12915_2021_1038_MOESM2_ESM.tif]

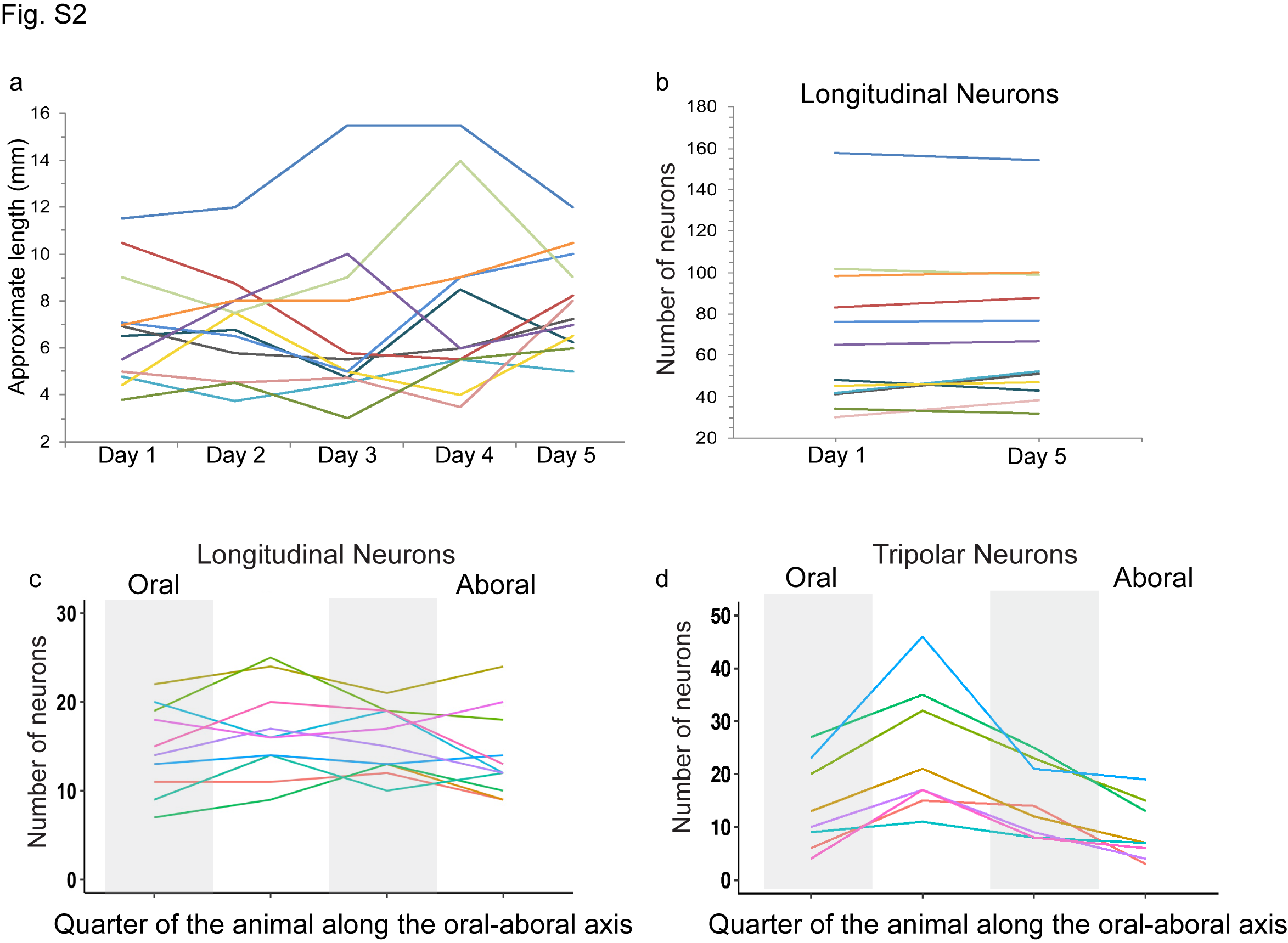

Supplement: Supplementary file 3 — Additional file 3: Fig. S2. Length measurements and neural quantifications in Nematostella. a The average variability in the measured length of the same animal over 5 consecutive days was 14% ± 3%. b The number of longitudinal neurons did not change over the same 5 day period in the same animals as A (t11 = -1.57, p = 0.145). c, d Quantification of the number of longitudinal and tripolar neurons along the oral-aboral axis in each of the 4 equal quarters. Gray and white bars distinguish the 4 quadrants. Longitudinal neurons are equally distributed along the oral-aboral axis (F3,45 = 1.18, p = 0.33, ηp2 = 0.73; n = 10), but tripolar neurons are not (F3,24 = 20.97, p < 0.001, ηp2 = 0.72; n = 8). Each line represents an individual animal. [file 12915_2021_1038_MOESM3_ESM.tif]

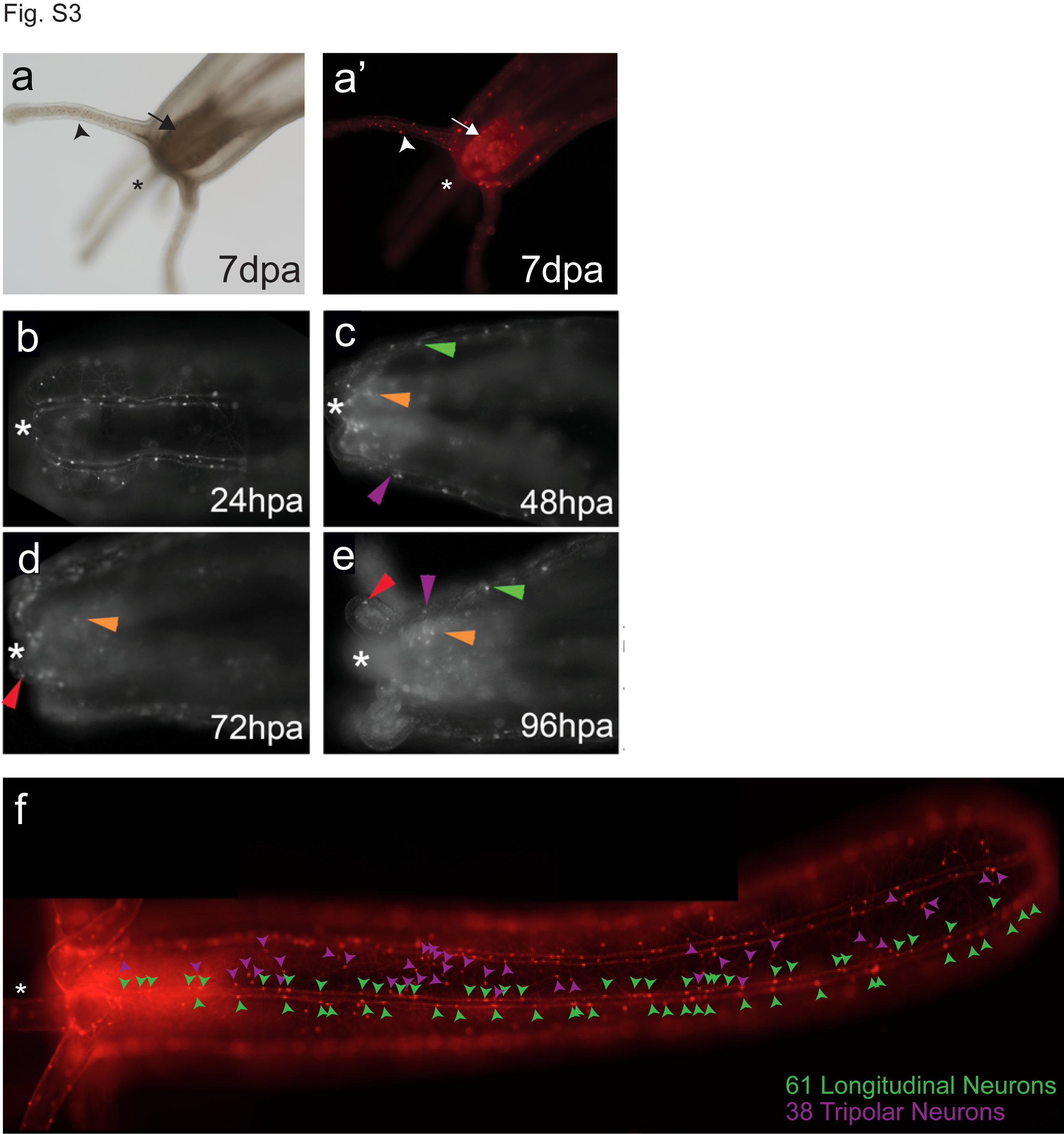

Supplement: Supplementary file 6 — Additional file 6: Fig. S3. Regeneration of oral structures and neurons in NvLWamide-like::mCherry animals following bisection along the oral-aboral axis. a Regenerated oral structures, including a mouth, pharynx (arrow), and tentacles (arrowhead) at 7dpa. a’ New neurons are observed in the regenerated oral structures, including pharyngeal (arrow) and tentacular (arrowhead) neurons. b Remnant fragment at 24hpa. No new structures were observed at this time point. c Tentacle buds were visible in the remnant fragment at 48hpa. Longitudinal (green arrowhead) and tripolar (purple arrowhead) neurons were observed. Regenerated pharyngeal neurons were also present in the regenerating pharynx (orange arrowhead). d Pharyngeal (orange arrowhead) and tentacular (red arrowhead) neurons were clearly visible in these regenerating structures by 72hpa. e Clearly regenerated pharyngeal (orange arrowhead) and tentacular (red arrowhead) neurons are seen at 96hpa. Longitudinal neurons (green arrowhead) and tripolar neurons (purple arrowhead) also populated the regenerated tissue, but whether these were regenerated neurons or from the remnant was undetermined. f Example of how longitudinal (green arrowheads) and tripolar (purple arrowheads) were quantified within a longitudinal track and radial segment, respectively. Note that for regeneration and feed-starve experiments, neuron counts were performed live under a dissection scope and not from images. Asterisks indicate oral opening. [file 12915_2021_1038_MOESM6_ESM.tif]

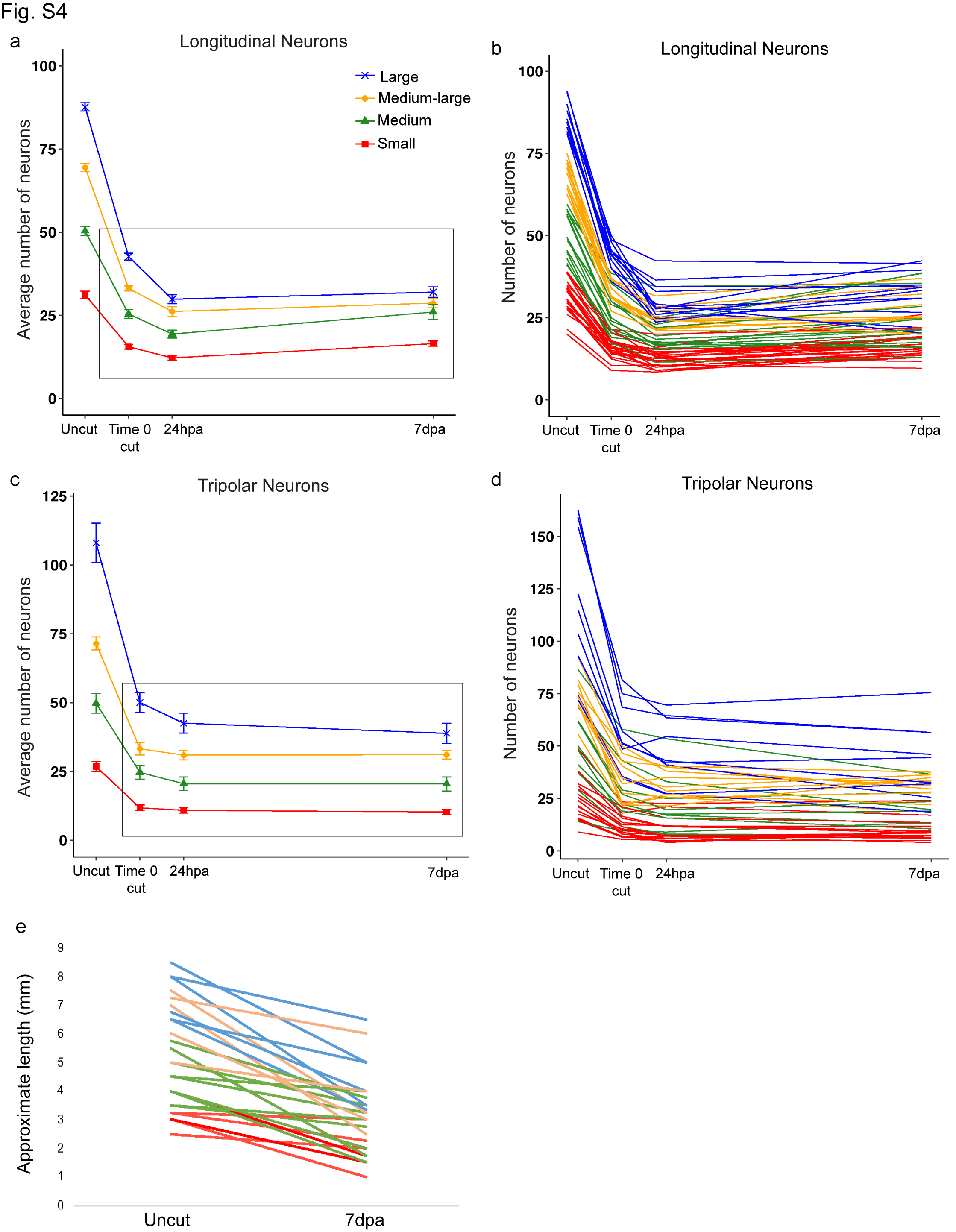

Supplement: Supplementary file 7 — Additional file 7: Fig. S4. Quantification of neuronal numbers during oral regeneration. a, c Quantification of mean longitudinal and tripolar neurons before amputation (uncut), immediately following bisection at the oral-aboral axis midline (time 0 cut), 24 hours post amputation (hpa), and at completion of regeneration 7 days post amputation (dpa). Black rectangles show time points included in analyses as seen in Fig. 4f & h. b, d Population data showing the time course for the average number of longitudinal neurons and tripolar neurons per 2-4 radial segments in individual regenerating animals bisected in half (n = 83 animals). Neuronal numbers did not return to levels present in the uncut animal. Data represented in b & d are the same dataset grouped by starting size and averaged in a & c and Fig. 4f & h. e Population data showing lengths for individual animals before they were amputated (uncut) and 7dpa. Regenerated animals never re-grew back to their original length (t30 = 8.65, p < 0.001; n = 31; see Fig. 4b for percent change by size). Small animals are represented in red, medium in green, medium-large in orange, and large in blue. Data points and bars represent means ± SEM. [file 12915_2021_1038_MOESM7_ESM.tif]

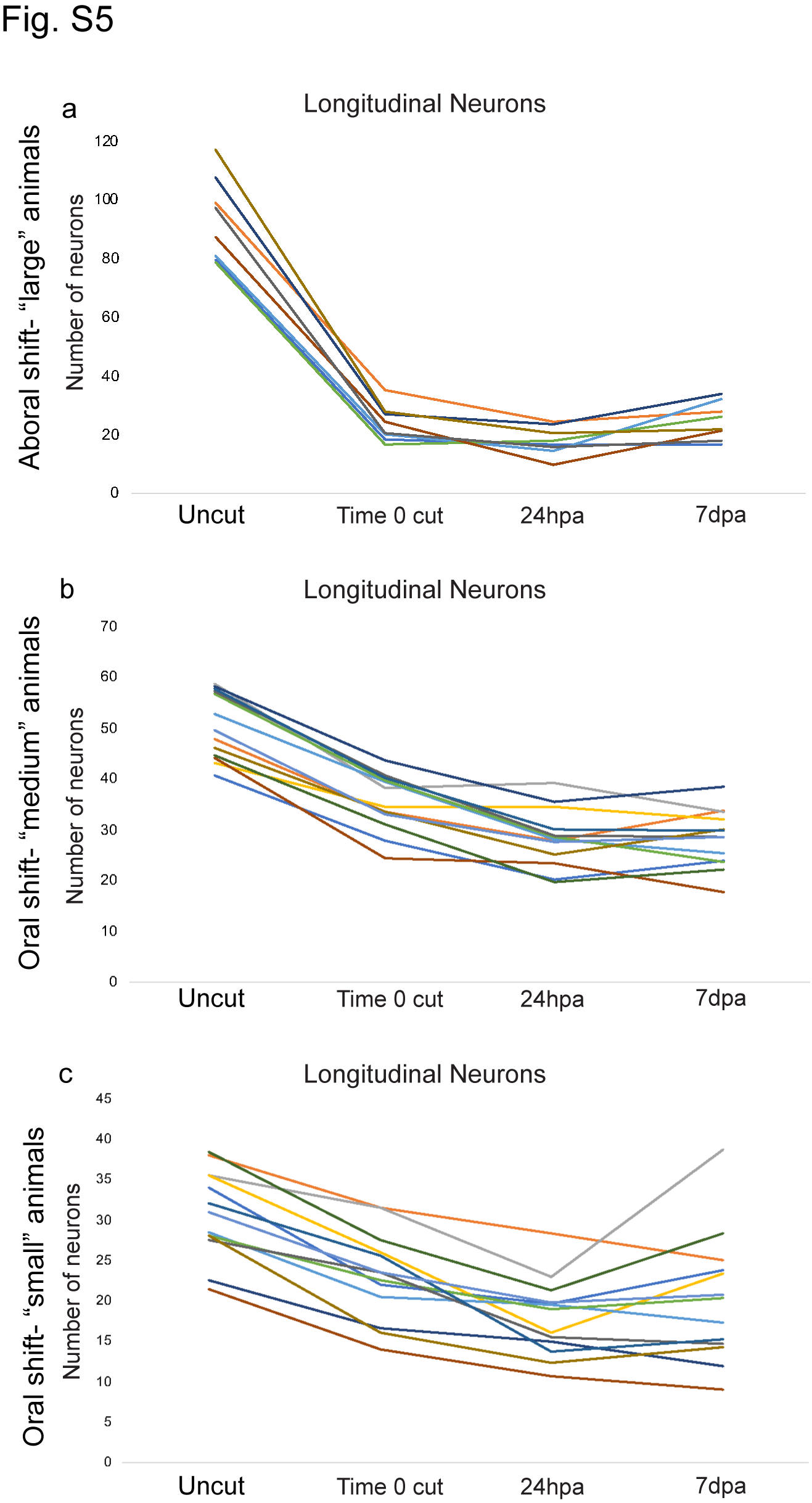

Supplement: Supplementary file 8 — Additional file 8: Fig. S5. Individual longitudinal neuron data from the shifted amputation site experiments. a Regenerates from an aborally shifted amputation site in large animals increased their longitudinal neurons back to similar numbers present at the time of bisection (n = 10). b Regenerates from orally shifted amputation sites in medium animals did not increase their longitudinal neurons (n = 13). c Regenerates from orally shifted amputation sites in small animals did not increase their longitudinal neurons (n = 13). Each line represents an individual animal. These data were used to determine the average responses shown in Fig. 5. See Additional file 15 for statistical analyses. [file 12915_2021_1038_MOESM8_ESM.tif]

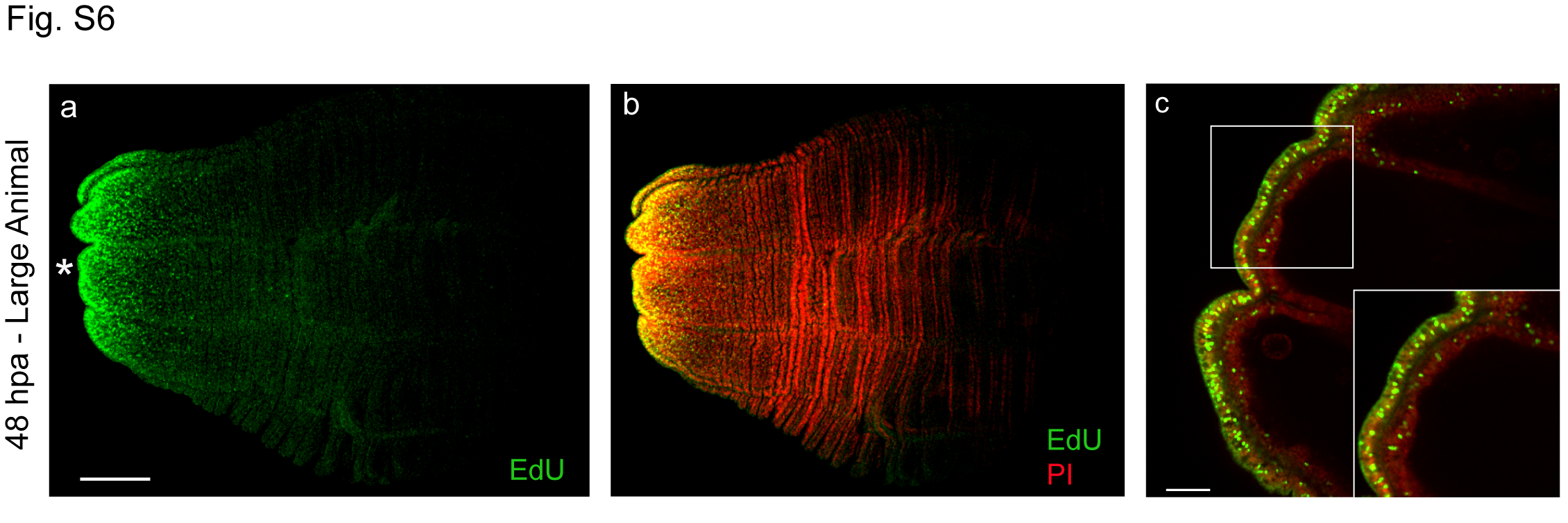

Supplement: Supplementary file 14 — Additional file 14: Fig. S6. Detection of proliferating cells in “large” Nematostella during oral regeneration. a EdU (green) is detected at 48hpa in the oral-most region of large regenerating animals bisected in half. b EdU overlaid with propidium iodide (PI, red) stain in the same animal as a, with yellow color showing co-expression. c Higher magnification image of the same animal, showing EdU detection in both the ectoderm and endoderm, suggesting both tissue layers proliferate during oral regeneration (n = 3 large animals). Asterisks indicate oral opening. Scale bars for a & b = 200μm; c= 50μm. [file 12915_2021_1038_MOESM14_ESM.tif]
