# Supplementary material for: Characterization of the dynamics and variability of neuronal subtype responses during growth, degrowth, and regeneration of Nematostella vectensis
Source: BMC Biol. 2021 May 18;19:104. doi: 10.1186/s12915-021-01038-9 (PMC8128482; doi:10.1186/s12915-021-01038-9)
Supplement: Supplementary file 15 — Additional file 15: Table S5. Supplemental ANOVA tables. [file 12915_2021_1038_MOESM15_ESM.pdf]

Table S5: Full ANOVA model results

Fig. 2: *Nematostella* neuronal subtypes scale with changes in size.
Mixed ANOVA analyses were performed for the data presented in Fig. 1c-e. The main effect of the repeated measure (observation time), between-subject factor (feeding regime), and the interaction effect (time x feeding) are reported for animal length (c), longitudinal neurons (d) and tripolar neurons (e). Main effects were interpreted within the context of any significant interaction effects. Bonferroni post-hoc testing was used to determine pairwise differences. S-F, starved then fed; F-S, fed then starved.

**c) Length**				
Factors	*Df*	*F*	*P*	η_p^2
• Observation time	2,34	6.63	**0.004**	0.28
• Feeding regime	1,17	1.02	0.326	0.06
• Time x Feeding	2,34	25.75	**< 0.001**	0.602
Pairwise comparisons:			*Mean difference*	*P*
(Feeding by Time)				
Time 0:	S-F vs. F-S		0.22	0.280
Feeding regime switch:	S-F vs. F-S		- 0.62	**0.003**
Week 14:	S-F vs. F-S		0.91	**0.002**
(Time by Feeding)				
S-F:	Time 0 vs. Feed switch		0.32	0.098
	Time 0 vs. Week 14		- 0.72	**0.002**
	Feed switch vs. Week 14		- 1.04	**< 0.001**
F-S:	Time 0 vs. Feed switch		- 0.52	**0.008**
	Time 0 vs. Week 14		- 0.03	1.00
	Feed switch vs. Week 14		0.49	**0.008**

**d) Number of longitudinal neurons**				
Factors	*Df*	*F*	*P*	η_p^2
• Observation time	2,36	9.91	**< 0.001**	0.36
• Feeding regime	1,18	0.52	0.82	0.003
• Time x Feeding	2,36	77.35	**< 0.001**	0.81
Pairwise comparisons:			*Mean difference*	*P*
(Feeding by Time)				
Time 0:	S-F vs. F-S		18.3	0.43
Feeding switch:	S-F vs. F-S		- 129.6	**0.001**
Week 14:	S-F vs. F-S		127.7	**< 0.001**
(Time by Feeding)				
Starved then fed:	Time 0 vs. Feed switch		44.6	**0.007**
	Time 0 vs. Week 14		- 100.3	**< 0.001**
	Feed switch vs. Week 14		- 144.8	**< 0.001**
Fed then starved	Time 0 vs. Feed switch		- 103.3	**< 0.001**
	Time 0 vs. Week 14		9.1	1.00
	Feed switch vs. Week 14		112.4	**< 0.001**

**e) Number of tripolar neurons**				
Factors	*Df*	*F*	*P*	η_p^2
• Observation time	2,36	20.69	**< 0.001**	0.54
• Feeding regime	1,8	2.09	0.19	0.21
• Time x Feeding	2,36	91.91	**< 0.001**	0.84

Pairwise comparisons:		*Mean difference*	*P*
(Feeding by Time)			
Time 0:	S-F vs. F-S	13.4	0.61
Feeding regime switch:	S-F vs. F-S	- 115.1	**< 0.001**
Week 14:	S-F vs. F-S	184.6	**< 0.001**
(Time by Feeding)			
S-F:	Time 0 vs. Feed switch	64.9	**0.03**
	Time 0 vs. Week 14	- 147.5	**< 0.001**
	Feed switch vs. Week 14	- 212.4	**< 0.001**
F-S:	Time 0 vs. Feed switch	- 63.6	**0.032**
	Time 0 vs. Week 14	23.7	0.70
	Feed switch vs. Week 14	87.3	**0.03**

Fig. 4: *NvLWamide-like* neuronal subtypes have differential responses during regeneration. Mixed ANOVA analyses were performed for the data presented in Fig. 4f & h. The main effect of the repeated measure (observation time), between-subject factor (starting size category), and the interaction effect (time x size) are reported for longitudinal (f) and tripolar neurons (h). Main effects were interpreted within the context of any significant interaction effects. Bonferroni post-hoc testing was used to determine pairwise differences. Greenhouse-Geisser corrected *F* statistics are reported for longitudinal and tripolar data sets (f, h), due to a lack of sphericity. Dpa, days post amputation; hpa, hours post amputation.

f) Regeneration of longitudinal neurons

Factors		*Df*	*F*	*P*	η_p^2
• Observation time		1.7,134.4	83.95	**< 0.001**	0.52
• Starting size		3,79	79.11	**< 0.001**	0.75
• Time x Size		5.1,134.4	13.46	**< 0.001**	0.34
Pairwise comparisons:				*Mean difference*	*P*
(Time by Size)					
Small:	Time 0 cut vs. 24 hpa			3.3	**< 0.001**
	Time 0 cut vs. 7 dpa			- 1.0	0.99
	24 hpa vs. 7 dpa			- 4.3	**< 0.001**
Medium:	Time 0 cut vs. 24 hpa			6.1	**< 0.001**
	Time 0 cut vs. 7 dpa			-0.6	1.00
	24 hpa vs. 7 dpa			- 6.6	**< 0.001**
Medium-large:	Time 0 cut vs. 24 hpa			6.9	**< 0.001**
	Time 0 cut vs. 7 dpa			4.4	**0.003**
	24 hpa vs. 7 dpa			- 2.5	0.25
Large:	Time 0 cut vs. 24 hpa			12.8	**< 0.001**
	Time 0 cut vs. 7 dpa			10.6	**< 0.001**
	24 hpa vs. 7 dpa			- 2.1	0.34
(Size by Time)					
Time 0 cut:	Small vs. Medium			- 9.9	**< 0.001**
	Small vs. Medium-large			- 17.5	**< 0.001**
	Small vs. Large			- 27.1	**< 0.001**
	Medium vs. Medium-large			- 7.7	**< 0.001**
	Medium vs. Large			- 17.2	**< 0.001**
	Medium-large vs. Large			- 9.5	**< 0.001**
24 hpa:	Small vs. Medium			- 7.2	**< 0.001**
	Small vs. Medium-large			-14.0	**< 0.001**
	Small vs. Large			- 17.6	**< 0.001**
	Medium vs. Medium-large			- 6.8	**< 0.001**
	Medium vs. Large			- 10.5	**< 0.001**
	Medium-large vs. Large			- 3.7	0.19
7 dpa:	Small vs. Medium			- 9.5	**< 0.001**
	Small vs. Medium-large			- 12.2	**< 0.001**
	Small vs. Large			- 15.5	**< 0.001**
	Medium vs. Medium-large			- 2.7	1.00
	Medium vs. Large			- 6.0	**0.05**
	Medium-large vs. Large			- 3.3	0.96

**h) Regeneration of tripolar neurons**				
Factors	*Df*	*F*	*P*	η_p^2
• Observation time	1.5,116.2	22.75	**< 0.001**	0.23
• Starting size	3,77	54.18	**< 0.001**	0.68
• Time x Size	4.5,116.2	3.93	**0.003**	0.13

Pairwise comparisons:		*Mean difference*	*P*
(Time by Size)			
Small:	Time 0 cut vs. 24 hpa	0.9	1.00
	Time 0 cut vs. 7 dpa	1.2	1.00
	24 hpa vs. 7 dpa	0.4	1.00
Medium:	Time 0 cut vs. 24 hpa	4.4	**0.001**
	Time 0 cut vs. 7 dpa	4.2	0.08
	24 hpa vs. 7 dpa	- 0.2	1.00
Medium-large:	Time 0 cut vs. 24 hpa	3.8	**0.013**
	Time 0 cut vs. 7 dpa	3.0	0.46
	24 hpa vs. 7 dpa	- 0.8	1.00
Large:	Time 0 cut vs. 24 hpa	6.8	**< 0.001**
	Time 0 cut vs. 7 dpa	10.2	**< 0.001**
	24 hpa vs. 7 dpa	3.4	0.055
(Size by Time)			
Time 0 cut:	Small vs. Medium	- 13.6	**< 0.001**
	Small vs. Medium-large	- 23.9	**< 0.001**
	Small vs. Large	- 38.3	**< 0.001**
	Medium vs. Medium-large	- 10.3	**0.03**
	Medium vs. Large	- 24.7	**< 0.001**
	Medium-large vs. Large	- 14.4	**0.001**
24 hpa:	Small vs. Medium	- 10.1	**0.003**
	Small vs. Medium-large	-21.1	**< 0.001**
	Small vs. Large	- 32.4	**< 0.001**
	Medium vs. Medium-large	- 11.0	**0.005**
	Medium vs. Large	- 22.3	**< 0.001**
	Medium-large vs. Large	- 11.3	**0.004**
7 dpa:	Small vs. Medium	- 10.6	**0.003**
	Small vs. Medium-large	- 22.2	**< 0.001**
	Small vs. Large	- 29.3	**< 0.001**
	Medium vs. Medium-large	- 11.6	**0.005**
	Medium vs. Large	- 18.7	**< 0.001**
	Medium-large vs. Large	- 7.1	0.22

Fig. 5: Differential regenerative responses of longitudinal neurons are partially dependent on the size of the remnant fragment. Repeated measure ANOVA analyses were performed for the data presented in Fig. 5c, e, g. Observation time served as the repeated measure for large animals with an aboral shift in cut site (c), medium animals with an oral shift in cut site (e), and small animals with an oral shift in cut site (g). Bonferroni post-hoc tests were used to evaluate pairwise differences when there was a significant main effect of observation time on the number of neurons observed. Dpa, days post amputation; hpa, hours post amputation.

**c) Regeneration of large animals with aboral shift in cut site**				
Factors	*Df*	*F*	*P*	η_p^2
• Observation time	2,14	5.58	**0.017**	0.44
Pairwise comparisons:			*Mean difference*	*P*
Time 0 cut vs. 24 hpa			5.8	**0.04**
Time 0 cut vs. 7 dpa			- 1.0	1.00
24 hpa vs. 7 dpa			- 6.9	**0.046**
**e) Regeneration of medium animals with oral shift in cut site**				
Factors	*Df*	*F*	*P*	η_p^2
• Observation time	2,24	21.67	**< 0.001**	0.64
Pairwise comparisons:			*Mean difference*	*P*
Time 0 cut vs. 24 hpa			7.0	**< 0.001**
Time 0 cut vs. 7 dpa			7.1	**0.001**
24 hpa vs. 7 dpa			0.05	1.00
**g) Regeneration of small animals with oral shift in cut site**				
Factors	*Df*	*F*	*P*	η_p^2
• Observation time	2,24	8.56	**0.002**	0.42
Pairwise comparisons:			*Mean difference*	*P*
Time 0 cut vs. 24 hpa			5.1	**< 0.001**
Time 0 cut vs. 7 dpa			2.9	0.12
24 hpa vs. 7 dpa			-2.2	0.47
